# The New Variation in the Promoter Region of *FLOWERING LOCUS T* Is Involved in Flowering in *Brassica rapa*

**DOI:** 10.3390/genes13071162

**Published:** 2022-06-27

**Authors:** Qingzhen Wei, Tianhua Hu, Xinfeng Xu, Zhen Tian, Chonglai Bao, Jinglei Wang, Hongtao Pang, Haijiao Hu, Yaqin Yan, Tongkun Liu, Wuhong Wang

**Affiliations:** 1 Institute of Vegetables Research, Zhejiang Academy of Agricultural Sciences, Hangzhou 310021, China; weiqz@zaas.ac.cn (Q.W.); huth@zaas.ac.cn (T.H.); baocl@zaas.ac.cn (C.B.); wangjinglei@zaas.ac.cn (J.W.); panghongtao@stu.zafu.edu.cn (H.P.); huhj@zaas.ac.cn (H.H.); yanyq@zaas.ac.cn (Y.Y.); 2 Engineering Research Center of Germplasm Enhancement and Utilization of Horticultural Crops, Ministry of Education, Nanjing 210095, China; 2020104061@stu.njau.edu.cn; 3 College of Ecology, Lishui University, Lishui 323000, China; tianz0326@lsu.edu.cn

**Keywords:** *B. rapa* ssp. *chinensis*, flowering time, quantitative trait locus, *FLOWERING LOCUS T*, promoter region

## Abstract

Flowering time is an important agronomic trait in *Brassica rapa* and has a wide range of variation. The change from vegetative to reproductive development is a major transition period, especially in flowering vegetable crops. In this study, two non-heading Chinese cabbage varieties with significantly different flowering times, Pak-choi (*B. rapa* var. *communis* Tesn et Lee) and Caitai (*B. rapa* var. *tsaitai* Hort.), were used to construct segregated F_2_ populations. The bulk-segregant approach coupled with whole genome re-sequencing was used for QTL sequencing (QTL-seq) analysis to map flowering time traits. The candidate genes controlling flowering time in *B. rapa* were predicted by homologous gene alignment and function annotation. The major-effect QTL *ft7.1* was detected on chromosome A07 of *B. rapa*, and the *FT* family gene *BrFT* was predicted as the candidate gene. Moreover, a new promoter regional difference of 1577 bp was revealed by analyzing the sequence of the *BrFT* gene. The promoter region activity analysis and divergent gene expression levels indicated that the difference in the promoter region may contribute to different flowering times. These findings provide insights into the mechanisms underlying the flowering time in *Brassica* and the candidate genes regulating flowering in production.

## 1. Introduction

The flowering characteristics of crops are important agronomic traits that are closely related to agricultural production. Flowering time has always been the focus of researchers and breeders, as it is useful for improving crop introduction and breeding. The transition from vegetative to reproductive growth is controlled by various physiological factors and genetic pathways [1]. For example, artificial lighting can affect the flowering of chrysanthemum and is conducive to year-round production [2]. In potatoes, early flowering reduces the yield and quality of vegetative organs [3]. Therefore, appropriately regulating flowering time is especially important to maximize reproductive success and seed production.

The molecular mechanisms underlying plant flowering have been extensively studied in a series of plants. In *Arabidopsis*, flowering involves six major pathways, including the photoperiod/circadian clock, vernalization, age, autonomy, ambient temperature, and gibberellin pathways [4,5]. More than 180 *Arabidopsis* genes are implicated in the flowering time networks, such as *FLOWERING LOCUS T* (*FT*), *CONSTANS* (*CO*), *FLOWERING LOCUS* *C* (*FLC*), and *FRI* (*FRIGIDA*) [6,7,8]. It is suggested that the transcriptional regulation of the florigen gene *FT* is crucial for integrating information from internal and external factors, i.e., age, the amount of gibberellic acid, and temperature [9]. *FT* plays pivotal roles in floral induction and encodes a long-distance floral signaling molecule that moves from the distal end of the leaf to the meristem [10,11,12,13]. The flowering time is generally controlled by quantitative trait loci (QTLs). *FT* homologs have been identified in many crops, including tomato [14], rice [15], soybean [16], onion [17], and cucumber [18].

The large genus *Brassica* comprises a diverse group of important oil, fodder, and vegetable crops. Cultivated varieties of diploid *B. rapa* exhibit highly diverse morphological and developmental traits. The flowering time in *B. rapa* is a significant developmental trait that is usually controlled by the temperature and/or photoperiod. Non-heading Chinese cabbage (*B. rapa* ssp. *chinensis*, 2n = 20; NHCC) belongs to the *B. rapa* non-heading Chinese cabbage subspecies, which includes five other variants [19]. The photoperiod response to changes in day length and the vernalization response to low temperatures are two major pathways that regulate the flowering time in *Arabidopsis thaliana* [9]. The vernalization pathway is considered a main flowering path and is highly divergent among different subgroups. Generally, the early flowering types, including the oil types and several Pak-choi cultivars, flower very early, even without vernalized conditions. Chinese cabbages and turnips are mostly late-flowering types that need long-term vernalization to promote flowering. A low temperature is needed for the flowering of NHCC, whose budding, bolting, and flowering time are affected by the site, temperature, and time of vernalization treatment [20]. Previous studies have mainly focused on the molecular mechanism of genes during bolting and flowering in NHCC. Researchers have confirmed that *MAF*, *TEM*, and *FLC* family genes play important roles in regulating NHCC bolting and flowering [20,21,22]. In addition, the bolting and flowering of NHCC is not only regulated by the vernalization pathway but is also affected by other factors. For example, previous studies have shown that photoperiodic flowering-related genes *BrCDF1*, *BcGI*, *BcFT*, etc. were involved in the flowering process of NHCC [23,24,25]. As a biennial vegetable, NHCC is usually sown in autumn and flowers in the next spring after overwintering. Because the young stems or leaves are the main consumed parts, early bolting and flowering can reduce the quality and yield of NHCC [22], directly affecting the economic benefits. Therefore, the research on candidate genes controlling bolting/flowering in NHCC has important practical significance for the regulation and selection of bolting-tolerant or late-bolting cultivars.

In this study, we conducted QTL-seq using two NHCC cultivars, wym-97 and cx-49, to identify the major QTLs controlling the flowering time of NHCC. Candidate genes were identified using QTL-seq and resequencing. Our study will provide a theoretical foundation for the development of elite genes and the regulation of NHCC flowering.

## 2. Materials and Methods

### 2.1. Plant Materials and Phenotypic Analysis

Two non-heading Chinese cabbage inbred lines (wym-97 and cx-49) with contrast flowering times were used to construct mapping populations in this study. The inbred line wym-97 (var. *communis* Tesn et Lee) is a late-flowering variety, and cx-49 (var. *tsaitai* Hort.) is an early flowering variety. Suzhouqing is a late-flowering cultivar of NHCC and used in promoter activity analysis. wym-97 and cx-49 were used as parent lines to generate the F_1_ (wym-97 × cx-49) plants. Two F_2_ populations were then generated by the self-pollination of F_1_ plants, i.e., 1-F_2_ and 2-F_2_. The 350 and 250 individuals of the 1-F_2_ and 2-F_2_ population were grown in the cities of Hangzhou (the Qiaosi experimental field, 120°36′ E, 30°37′ N) and Lishui (the Bihuzhen experimental field, 119°79′ E, 28°35′ N) for phenotypic statistics of their respective flowering traits. The seeds were planted in early November, and the flowering time, which refers to the days from sowing to flowering, was treated as the target trait in this study. The statistical analysis on phenotypic data was performed using Microsoft Excel 2019 (Microsoft, Seattle, Washington, DC, USA).

### 2.2. Sample Collection and Bulk Construction

According to the phenotypic data, the individuals with an extremely early or late flowering time were determined. Young leaves of selected extreme individuals were collected, frozen in liquid nitrogen, and immediately stored in a −80 °C freezer. Sample DNA was extracted using the modified CTAB method [26]. The purity and integrity were detected using agarose gels. The concentration was then detected using a Nanodrop to ensure that the DNA could be used to construct sequencing libraries. Equal amounts of DNA were pooled to form four DNA pools, namely, E1-bulk (40 early flowering individuals in the 1-F_2_ population), L1-bulk (40 late-flowering individuals in the 1-F_2_ population), E2-bulk (30 early flowering individuals in the 2-F_2_ population), and L2-bulk (30 late-flowering individuals in the 2-F_2_ population). Finally, the two parent lines (wym-97 and cx-49) and four extreme bulked pools were sequenced using an Illumina HiSeq 4000 PE150 (Illumina, Inc., San Diego, CA, USA).

### 2.3. QTL-Seq Analysis and the Prediction of Candidate Genes

The Illumina TruSeq was used to construct the sequencing libraries, and BWA software [27] was used to compare the sequenced reads with the reference genome of NHCC [19] after filtering low-quality reads. GATK software was used to detect SNP information, and ANNOVAR was employed to perform SNP annotation. After filtering, the SNP index distribution of the four bulks was obtained in a 2 Mb window and a 50 kb step size. The Δ(SNP-index) value was calculated by subtracting the SNP index of L1/L2-bulks from that of E1/E2-bulks, respectively. The regions above the yellow thresholds (*p* < 0.01) were regarded as candidate QTLs. The candidate genes were then predicted by delineation of the Δ(SNP-index) peak region and the functional annotation of flowering homologous genes. The peak region is the top 15% of the sliding window points sorted according to the Δ(SNP-index) value within candidate QTLs.

### 2.4. Comparison of Candidate Gene Promoter Activities

The promoters of the candidate gene in wym-97, cx-49 and suzhouqing were cloned using primers QCFT-pF6/pR1 and then recombined into the vector pGreenⅡ-0800-LUC. The constructed vector and control (empty vector) were transformed into Agrobacterium GV3101. The inocula (OD600 = 0.8) were injected into tobacco leaves, protected from light overnight, and then cultured under normal light conditions for 72 h. Fluorescein potassium salt (Yeasen, Shanghai, China) was then injected and placed in the dark for 5 min. The images were observed and photographed using an in vivo plant imaging system (LB985 Night SHADE, Stuttgart, Germany).

### 2.5. Quantitative Verification

The quantitative experiment was conducted by the parent lines wym-97 and cx-49. After germination (20 August 2020), the seeds were sown in the substrate (peat soil:perlite:vermiculite = 1:1:1) and placed in an artificial climate room for cultivation at a temperature of 22 °C/18 °C (day/night), a light/dark ratio of 16 h/8 h (day/night), and a relative humidity of 60%. After 30 days, the tender leaves were sampled for quantitative validation experiments of the candidate genes.

### 2.6. RNA Extraction, cDNA Synthesis, and qRT-PCR Analysis

An amount of 0.1 g of tender leaves was ground into a powder for the extraction of total RNA (Trizol Reagent). Subsequently, 1 μg of RNA was used to synthesize a 20 μL cDNA system using the Prime-Script™ RT Reagent Kit with gDNA Eraser (TAKARA, Kusatsu, Japan). The total reaction system included 10 μL of SYBR Premix (2×), 1 μL of cDNA, 2 μL of primers (10 μM), and 7 μL of ddH_2_O. The qRT-PCR program was carried out using the Bio-Rad iCycler Real-Time PCR Detection System (Bio-Rad, Hercules, CA, USA), with pre-denaturation at 95 °C for 1 min, 40 cycles of denaturation at 95 °C for 10 s, annealing at 56 °C for 30 s, and extension at 72 °C for 30 s. Primer pairs were designed using Primer Premier 5.0 and are listed in Supplementary Table S1. The *Bcpp2* gene was used as an internal standard for normalizing the gene expression data. The relative expression levels were calculated using the 2^−ΔΔCT^ method [28] and visualized using Graphpad prism (vs. 8.4.3). *p* value = 0.05 means a significant difference (indicated by *); *p* value = 0.01 means a very significant difference (indicated by **).

## 3. Results

### 3.1. Phenotypic Analysis of the Flowering Time Characteristics

**Figure 1:**
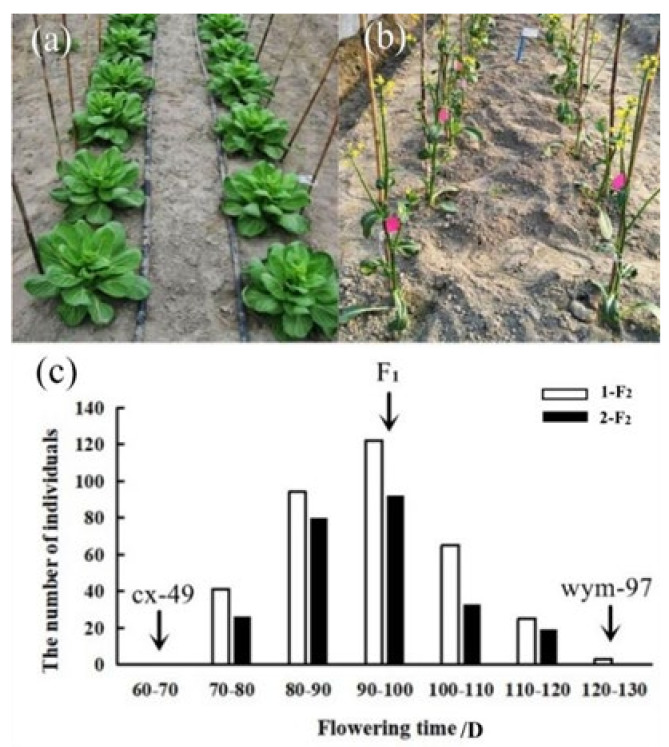
The flowering time characteristics and frequency distribution of the parent lines, F_1_ and the F_2_ populations. (**a**,**b**) The performance of the parent lines (80 d after sowing). (**c**) The frequency distribution of the flowering time of the cx-49, wym-97, F_1_, 1-F_2_, and 2-F_2_ populations. The flowering time of the two parents and F_1_ is indicated by arrows.

The flowering time was respectively measured from sowing to flowering in the spring of 2020 at Hangzhou and Lishui. A wide range of variation in flowering time was observed among the F_2_ populations derived from the cross between wym-97 and cx-49. The inbred line wym-97 (var. *communis* Tesn et Lee) is a late-flowering variety, where the leaves and petioles are the main consuming organs. In contrast, cx-49 (var. *tsaitai* Hort.) is an early flowering variety, where the scape (pedicel) is the main consuming organ. Two F_2_ populations were generated by the self-pollination of F_1_ individuals, including the 1-F_2_ population with 350 individuals and the 2-F_2_ population with 250 individuals. In the Hangzhou area, the average flowering time of wym-97 was 128 d, whereas that of cx-49 was 65 d, which was 63 d earlier than that of wym-97. The average flowering duration of the F_2_ plants was 92.7 d. The earliest flowering was 74 d, and the latest one was 120 d. In Lishui, the average flowering time of wym-97 was 120 d while that of cx-49 was 61 d, so the average flowering time of wym-97 was 59 d later than that of cx-49 at Lishui. The average flowering time of the F_2_ plants was 91.5 d. The earliest flowering time was 70 d, and the latest flowering time was 118 d. The flowering times of the F_2_ materials are presented in a normal distribution (Figure 1, Table 1), which is consistent with the quantitative trait inheritance characteristics. The results indicate that flowering time is a quantitative trait regulated by multiple loci.

### 3.2. Detection of QTLs

According to the phenotypic data, four bulked pools of F_2_ individuals (two pools for each F_2_ population) with extremely early or late flowering times and the two parental lines were subject to Illumina high-throughput sequencing. A total of 953,593,154 bp clean reads were generated, and the Q30 of each sample was above 90% (Table 2). The average sequencing depths of wym-97 and cx-49 were 28.71× and 29.50×, respectively, whereas the sequencing depths of the four pools were 74.54%, 69.76%, 61.87%, and 63.86%, respectively. The genome coverage ranged from 89.34% to 95.90% (at least one base coverage). The quality of the sequencing data was appropriate to satisfy the requirements of QTL-seq analysis.

**Figure 2:**
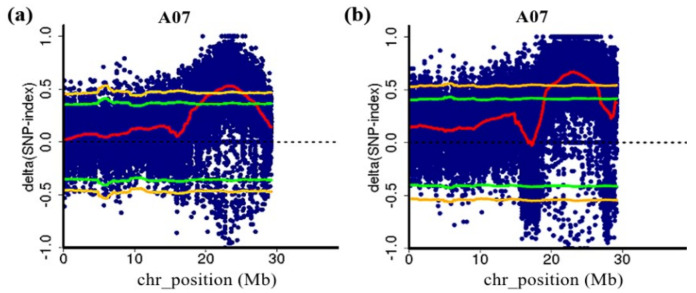
The major QTL *ft7.1* for the flowering time detected by QTL-seq. (**a**) QTL-seq analysis of chromosome 7 in the 1-F_2_ population; (**b**) QTL-seq analysis of chromosome 7 in the 2-F_2_ population. The blue dots are the Δ(SNP-index) of each SNP, and the red lines are the Δ(SNP-index) fitting lines, Δ(SNP-index) plot of the E and L bulks with confidence intervals under the null hypothesis of no QTLs (green, *p* < 0.05; yellow, *p* < 0.01).

QTL-seq analysis was performed using the genome sequence of non-heading Chinese cabbage as the reference genome [19]. The results showed that two QTLs related to flowering time were detected using two F_2_ populations, which were both located on chromosome 7 of NHCC (Figure 2). The major-effect QTLs were determined according to a 99% threshold line. In the 1-F_2_ population, one QTL (*FT7.1*) was detected with a QTL interval of 21.10–25.25 Mb and a range size of 4.15 Mb. The Δ(SNP-index) value of *FT7.1* was 0.463–0.530 (Supplementary Table S2). In the 2-F_2_ population, one QTL (*FT7.2*) was also identified. The interval was 20.10–26.15 Mb with a range size of 6.05 Mb. The maximum Δ(SNP-index) value was 0.671, and the minimum was 0.549 (Supplementary Table S3). Both QTLs were on chromosome 7, which shared an overlapping interval designated *ft7.1* (21.10–25.25 Mb). Therefore, the major QTL *ft7.1* was used for the subsequent analysis.

### 3.3. The Prediction of Candidate Genes

The Δ(SNP-index) values in candidate regions were analyzed to further predict the candidate genes within the two QTLs *FT7.1* and *FT7.2*. A 2 Mb window and a 50 Kb step size were used in the QTL-seq analysis. There were 84 and 122 sliding window points in the 4.15 and 6.05 Mb intervals, respectively, and the sliding window points with Δ(SNP-index) values in the top 15% delineated a reduced peak target region (Supplementary Table S4). The peak value of *FT7.1* ranged from 22.90 to 23.75 Mb, and the Δ(SNP-index) values ranged from 0.527 to 0.530. The peak value and the Δ(SNP-index) values of *FT7.2* ranged from 22.65 to 23.75 Mb and from 0.660 to 0.671, respectively (Table 3 and Supplementary Table S4). Thus, we obtained the peak region of 22.65–23.55 Mb shared by the two QTLs. Meanwhile, a homology comparison was also performed with the flowering homologous genes in *A. thaliana* in the shared region of *FT7.1* and *FT7.2*. There are 8 candidate genes related to flowering in the major QTL *ft7.1* (Table 4), among which *BraC07g031540* was located in the peak region of 22.65–23.55 Mb shared by the two QTLs. Based on the above results, the *BraC07g031540* (designated *BrFT*) is likely to be a candidate gene controlling flowering time in our materials.

### 3.4. Sequence Polymorphism Analysis of the BrFT Gene

**Figure 3:**
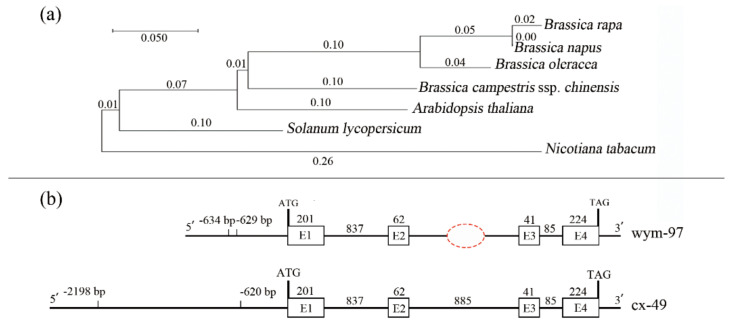
Phylogenetic analysis of the *FT* genes and comparison of *BrFT* gene variations between two parent lines. (**a**) Phylogenetic tree of the *FT* genes from different species. (**b**) The comparison of *BrFT* gene variations between the two parent lines (cx-49 and wym-97).

The phylogenetic relationships of the *BrFT* gene among the *Brassica* and other plant species were investigated. A phylogenetic tree, including five Brassica species, tomato, and tobacco, was constructed using MEGA X (Figure 3a).

**Figure 4:**
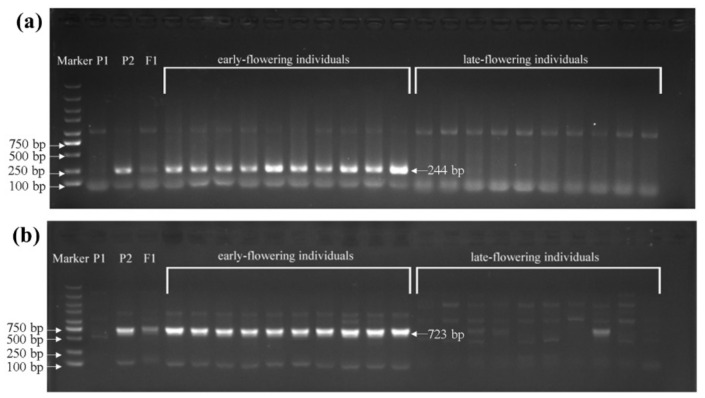
The variation detection of the *BrFT* gene in extreme individuals in the 1-F_2_ and 2-F_2_ populations. P_1_, wym-97; P_2_, cx-49. (**a**) The variation detection in the promoter region using primer pFT2F/2R. (**b**) The variant detection in the intronic region using primer BrFTF5-1/R5-1.

To identify sequence variations in the *BrFT* gene between the two parents, genomic DNAs of wym-97 and cx-49 were used to design primers to amplify the *BrFT* gene. The cx-49 *BrFT* gene was successfully cloned; however, no product was amplified using the genomic DNA of wym-97. Additional segmented primer sets were designed to amplify the genomic sequence of *BrFT* in wym-97. A new variation in the promoter region was found by amplifying and sequencing the upstream promoter region of the *BrFT* gene. Compared with wym-97, there was a 1577 bp insertion in the promoter region of cx-49 (Figure 3b). In order to facilitate further identification of the extreme individual plants in the F_2_ population using molecular markers, a pair of primer pFT2F/2R was developed to detect the variation in the promoter region. The forward primer of the primer sequence was in front of the cx-49 promoter insertion fragment, and the reverse primer was within the cx-49 promoter insertion fragment, with a target amplified fragment of only 244 bp. We also used the primers to compare the results of the parents and 140 extremely early or late-flowering individual plants in the 1-F_2_ and 2-F_2_ populations. The results showed that the same fragment insertion as the early flowering parent cx-49 was found in the promoter region of the early flowering lines (Figure 4a). All late-flowering individuals were the same as wym-97. Thereby, the variations in the *BrFT* promoter region are reliable and the primers can be used for the marker-assisted screening of flowering time.

We also found that a stretch of sequence may be inserted between the 259 and 640 bp region of the intron 2 in wym-97, which resulted in failed PCR amplification. Thus, we designed the primer BrFTF5-1/R5-1 at the insertion site, the forward primer of which is in front of the insertion point and the reverse primer of which is behind the insertion point. Nonetheless, the primer could amplify a DNA fragment with 723 bp in length in cx-49 while wym-97 had no amplification product (Figure 4b). Subsequently, 140 individual plants of the 1-F_2_ and 2-F_2_ populations flowering extremely early and extremely late were used to verify the primers. The results showed that all early flowering individuals amplified the same bands as the early flowering parent cx-49. All late-flowering individuals showed the same diffuse bands as the late-flowering parent wym-97.

### 3.5. Verification of the Candidate Gene

**Figure 5:**
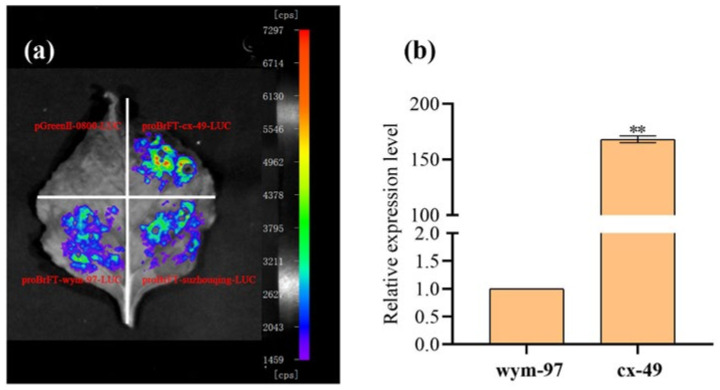
The verification of candidate gene *BrFT*. (**a**) The detection of promoter activities. (**b**) The expression level of *BrFT* in parent lines. ** indicated a very significant difference (*p* value = 0.01).

The same primer QCFT-pF6/QCFT-pR1 was used to clone the promoters of the *BrFT* gene in cx-49, wym-97, and the late-flowering cultivar suzhouqing. The promoter regions with lengths of 2342, 764, and 1374 bp were obtained using proBrFT-suzhouqing-LUC as a control. The comparative promoter activity results indicated that the fluorescence value of proBrFT-cx-49-LUC was higher than that of proBrFT-wym-97-LUC and proBrFT-suzhouqing-LUC. Therefore, the promoter activity of cx-49 is higher than that of wym-97. We speculated that the insertion in the promoter region of the early flowering parent cx-49 may activate the transcription of *BrFT*, thereby promoting the expression of *BrFT* in cx-49 (Figure 5a). The expression of the *BrFT* gene in the two parents was also analyzed (Figure 5b). We found that the expression level of *BrFT* in cx-49 was significantly higher than that in wym-97, indicating that the transcription of *BrFT* was more active in the early flowering parent cx-49.

## 4. Discussion

**Figure 6:**
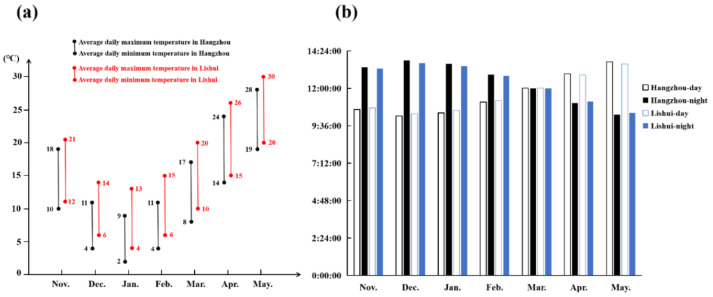
Statistics of temperature and light in the cultivation season. (**a**) The statistics of temperature during the cultivation season in Hangzhou and Lishui; (**b**) the statistics of the light condition in Hangzhou and Lishui.

Plant flowering is affected by many factors, among which temperature and light play essential roles [9]. Vernalization pathways are extensive flowering pathways in crucifers, whose flowering requires a low temperature [20]. Nevertheless, temperature is not the only factor that affects the flowering of crucifers. NHCC belongs to the *Brassica* non-heading Chinese cabbage subspecies, which also includes Pak-choi (var. *communis* Tesn et Lee), Caitai (var. *tsaitai* Hort.), Fenniecai (var. *multiceps* Hort.), Taicai (var. *taitsai* Hort.), and Tacai (var. *rosularis* Tsen et Lee) [19]. NHCC originated from the Taihu Lake of the Lower Yangtze River in China. Research on the flowering of NHCC showed that, after low-temperature treatment, long-day conditions can promote the flowering process [29]. In this study, the flowering time of NHCC was nearly a week later under a relatively low temperature environment at Hangzhou compared to Lishui. During the cultivation, the relatively high temperature and long daytime at Lishui could promote the flowering time of NHCC (Figure 6). Combined with previous studies, we propose that a low temperature can promote the flowering of NHCC, but the effect of light, especially after low-temperature treatment, on the flowering of NHCC is also important.

There are several important stages during the process of plant flowering. Therefore, different target traits have been used to determine flowering traits. Rosental et al. [30] mapped the QTLs affecting lettuce bolting/flowering under four environmental conditions. In wheat, the QTLs associated with flowering were identified by flowering and maturity time [31]. Liu et al. [32] detected the QTLs that controlled bolting in Chinese cabbage and studied the effect of epistasis and locus environment interactions. The bolting and flowering time were measured to identify the QTLs that regulate flowering in radish [33]. In this study, flowering time was used as the target trait and similar mapping results in different years were obtained, and the flowering characteristics of NHCC could be measured.

Many genes are reported to be involved in the flowering time networks [5], including *FT*, *FLC*, *CO*, *GIGANTEA* (*GI*), *FLAVIN KELCH F BOX 1* (*FKF1*), and *FRI* (*FRIGIDA*) [6,7,8]. The floral integrator gene *FT* encodes a long-distance systemic signal that moves from the leaves to the shoot meristem through the phloem [10,11,13] while CO is involved in the photoperiod pathway that activates the transcription of *FT*. The high expression of *FT* leads to early flowering in plants. FLC takes functions in the vernalization pathway by encoding an MADS-box transcription factor that represses the flowering time [34,35,36,37]. The FLC protein directly binds to the promoter region of the *FT* gene to repress expression and delay plant flowering [38,39]. In the shoot apical meristem, FT interacts with the bZIP transcription factor FD, forming a FT/FD heterodimer complex, which activates expression of the floral meristem identity genes, and finally results in the initiation of flower bud development [12,13,40,41,42]. Due to the whole-genome triplication event, there are multiple copies of flowering-time-controlling genes in the *B. rapa* genome, including four *FLC*, three *VRN1*, and two *FT* copies according to BRAD.

Previous studies have identified QTLs associated with flowering time in various crops [18,43,44,45]. Combined with QTL-seq and traditional mapping, the major QTL *Ef2.1* that regulates flowering time was also identified using the populations constructed by broccoli × cabbage. According to the functional annotation information, the candidate gene was predicted to be *BolGRF6* [46]. Gao et al. [47] used map-based cloning to identify the major QTL *DTH7* that regulates flowering time in rice, which encodes the PRR family gene *OsPRR37*. In cucumber, a major locus on flowering time in ‘Xishuangbanna’ cucumber was identified on chromosome 1 through traditional mapping and QTL-seq. The candidate gene *CsaNFYA1* and its network controlling flowering were analyzed [48]. Several QTL mapping studies have been reported on flowering time in *B. rapa* and have identified QTL regions where the *FLC* genes were predicted as candidate genes [44,49,50]. In oilseed-type *B. rapa*, QTL mapping and transcriptome analysis using ‘Chiifu’ and ‘LP08′ as parental materials indicated that *BrFLC2* is a candidate gene for rapid flowering in the early flowering cultivar ‘LP08′ [51]. A major QTL on chromosome A02 was detected in several *B. rapa* populations with *BrFLC2* as the candidate gene across different locations and seasons [52]. However, the research on bolting and flowering in NHCC has mainly focused on analyses of the molecular mechanisms of related genes. Moreover, due to the limitation of genomic data, previous research has mainly been based on the *B. rapa* genome data (BRAD; http://brassicadb.org (accessed on 9 March 2021). It is proposed that the strong effect of *FLC* on flowering time may obscure the effect of genes in the same pathway or other pathways [45]. In this study, we conducted a genetic mapping of QTLs controlling flowering time in NHCC through QTL-seq and identified the major QTL *ft7.1* on chromosome 7. The latest NHCC genome version was used as the reference genome [19] in QTL-seq analysis, which improved the accuracy of genetic mapping. Previous studies reported two major QTLs (*Flt1* and *Flt2*) controlling the budding time of NHCC using RIL populations that derived from non-vernalization parents [45]. The two genes *Bra022475* and *Bra004117* in the Chinese cabbage genome were detected as *A. thaliana FT* homologs, which were designated as *BrFT1* and *BrFT2*, respectively. However, there may be functional redundancy in the two *FT* paralogues in *B. rapa*. The *BrFT2* was considered as the candidate gene for flowering time in the RIL population. In this study, the *A. thaliana FT* homolog *BraC07g031540* in the NHCC genome was identified as the candidate gene in the overlapping QTL region, which was designated as *BrFT*. Sequence alignment analysis revealed a high homology between *Bra004117* and *BraC07g031540*. Despite the similar transposon insertion observed in *BrFT* (Figure 3) and *BrFT2* [45], we found a new insertion 1577 bp in length in the promoter region of *BrFT* (Figure 3), which was associated with delayed flowering in *B. rapa*. Nonetheless, *Bra004117* and *BraC07g031540* had the same CDS coding region and encoded protein sequences. Thereby, this gene plays a predominant role in controlling flowering time in populations with contrasting flowering habits and could be an excellent target for the improvement of bolting and flowering in *B. rapa* cultivation. However, multiple genes together form a complex network that regulates plant flowering. This study lays a theoretical foundation for the location of the flowering candidate genes of non-heading Chinese cabbage and a rational method for regulating its flowering in production.

## 5. Conclusions

In this study, two NHCC varieties with significantly different flowering times were used to construct two F_2_ populations for QTL-seq analysis. One major-effect QTL *ft7.1* was detected on chromosome 7 and *BrFT* was predicted as the candidate gene. We found a new promoter regional difference by analyzing the *BrFT* gene sequence in the genomes of two parents and the extreme F_2_ individuals. We propose that the differences in the promoter region may lead to divergent flowering times and that the *BrFT* gene is likely to be the main gene responsible for flowering differences in *B. rapa*.

## Figures and Tables

**Table 1:** Phenotypic variation in the flowering time of two F_2_ populations.

Place	Flowering-Time of Parents and F_1_ (Days)			Flowering-Time of F_2_ Populations (Days)			
	wym-97	cx-49	F_1_	Range	Mean ± SD	Kurtosis	Skewness
Hangzhou	128 ± 0.42	65 ± 0.39	96 ± 0.51	74–120	92.7 ± 0.59	−0.63	0.26
Lishui	120 ± 0.47	61 ± 0.36	91 ± 0.49	70–118	91.5 ± 0.63	−0.2	0.43

**Table 2:** Statistics of the sequencing data for the parents and extreme pools.

Sample Name	Clean_Base	Q30 (%)	Mapped (%)	Average Depth	Cov_Ratio_1X (%)
wym-97	83,327,924	93.46	97.60	28.71	89.34
cx-49	83,659,410	93.12	97.67	29.50	90.11
E1-bulk	233,767,054	91.46	97.56	74.54	94.98
L1-bulk	227,951,542	90.91	97.38	69.76	94.91
E2-bulk	159,912,040	90.65	97.59	61.87	95.81
L2-bulk	164,975,184	90.68	97.69	63.86	95.90

**Table 3:** Summary of QTLs detected for the flowering time with QTL-seq.

Population	QTL	Chr.	Chr. Position (Mb)	Interval (Mb)	Δ(SNP-Index) Range	The Peak Area (Mb)	Δ(SNP-Index) Range of the Top 15%
1-F_2_	*FT7.1*	A07	21.1–25.25	4.15	0.463–0.530	22.90–23.75	0.527–0.530
2-F_2_	*FT7.2*	A07	20.1–26.15	6.05	0.549–0.671	22.65–23.55	0.660–0.671

**Table 4:** The candidate genes related to flowering in the QTL *ft7.1*.

Gene ID	Chr.	Position		Gene Name	Homologous in *Arabidopsis*
*BraC07g025350.1*	A07	20019254	20020489	*GID1A* (GA INSENSITIVE DWARF1A)	*AT3G05120*
*BraC07g029460.1*	A07	22187954	22190945	*Cstf64* (CLEAVAGE STIMULATION FACTOR 64)	*AT1G71800*
*BraC07g030260.1*	A07	22638402	22640432	*CDF5* (CYCLING DOF FACTOR 5)	*AT1G69570*
*BraC07g030410.1*	A07	22738747	22742934	*AP1* (APETALA1)	*AT1G69120*
*BraC07g031540.1*	A07	23423378	23425730	*FT* (*FLOWERING LOCUS T*)	*AT1G65480*
*BraC07g034100.1*	A07	24821412	24825813	*AP1* (APETALA1)	*AT1G69120*
*BraC07g034480.1*	A07	25040930	25042186	*CDF5* (CYCLING DOF FACTOR 5)	*AT1G69570*
*BraC07g034690.1*	A07	25212811	25214341	*UBC1* (UBIQUITIN CONJUGATING ENZYME 1)	*AT1G14400*

## Data Availability

Raw sequence reads have been submitted to the NCBI Sequence Read Archive under the accession number PRJNA836239.

## Supplementary Materials

The following supporting information can be downloaded at: https://www.mdpi.com/article/10.3390/genes13071162/s1. Table S1: Summary of primer sequence information. Table S2: The Δ(SNP-index) value on Chromosome 7 in the 1-F2 population. Table S3: The Δ(SNP-index) value on Chromosome 7 in the 2-F2 population. Table S4: The Δ(SNP-index) value of the sliding window position of QTLs on Chromosome 7.
